# Protective Effect of Genistein against Compound 48/80 Induced Anaphylactoid Shock via Inhibiting MAS Related G Protein-Coupled Receptor X2 (MRGPRX2)

**DOI:** 10.3390/molecules25051028

**Published:** 2020-02-25

**Authors:** Mukesh Kumar, Kailash Singh, Karthi Duraisamy, Ahmed A. Allam, Jamaan Ajarem, Billy Kwok Chong CHOW

**Affiliations:** 1 School of Biological Sciences, The University of Hong Kong, Pokfulam Road, Hong Kong, China; mkumar@hku.hk (M.K.); kailash@connect.hku.hk (K.S.); h1258159@connect.hku.hk (K.D.); 2 Department of Zoology, Faculty of Science, Beni-Suef University, Beni-Suef 62511, Egypt; aallam@ksu.edu.sa; 3 Department of Zoology, College of Science, King Saud University, Riyadh 11451, Saudi Arabia; jajarem@KSU.EDU.SA

**Keywords:** anaphylactoid shock, mast cells, allergy, MRGPRX2

## Abstract

Anaphylactoid shock is a fatal hypersensitivity response caused by non-IgE mediated mast cell activation. These reactions are mediated by a family of G protein-coupled receptors (GPCRs) known as Mas related GPCRX2 (MRGPRX2). Several US FDA approved drugs which are used in day to day life have been reported to cause anaphylactoid shock. Surprisingly, no therapeutic drugs are available which can directly target MRGPRX2 for treatment of anaphylactoid shock. Genistein is a non-steroidal polyphenol known for its diverse physiological and pharmacological activities. In recent studies, Genistein has been reported for its anti-inflammatory activity on mast cells. However, the effects and mechanistic pathways of Genistein on anaphylactoid reaction remain unknown. In the present study, we designed a battery of in-vitro, in-silico and in-vivo experiments to evaluate the anti-anaphylactoid activity of Genistein in order to understand the possible molecular mechanisms of its action. The in-vitro results demonstrated the inhibitory activity of Genistein on MRGPRX2 activation. Further, a mouse model of anaphylactoid shock was used to evaluate the inhibitory activity of Genistein on blood vessel leakage and hind paw edema. Taken together, our findings have demonstrated a therapeutic potential of Genistein as a lead compound in the treatment of anaphylactoid shock via MRGPRX2.

## 1. Introduction

Anaphylaxis is a type-I IgE mediated allergic reaction caused by the activation of mast cells, whereas anaphylactoid reactions or pseudo-allergic reactions are non-immunologic sudden onset reactions mediated through the non-IgE pathway. The clinical symptoms of anaphylactoid shock, however, are similar to anaphylaxis [1]. Activation of mast cells by allergen or drugs causes a profound release of inflammatory mediators, which leads to anaphylactoid shock [2]. The clinical manifestations of anaphylactoid reaction are similar and indistinguishable from anaphylaxis, and sometimes even more severe, leading to cardiovascular collapse and death [1,3]. The exact incident rate of anaphylactoid reaction is difficult to be established, as many of them are not diagnosed or reported. Moreover, the estimated frequency varies considerably between epidemiological studies from different countries [4,5,6], while studies conducted over the last years provided an incident rate in the range of 40–500 per million persons per year [7,8,9,10,11]. 

Mast cells or mastocytes are one of the immune cells that contain many granules rich in histamine, heparin and inflammatory cytokines and are located primarily at sites exposed to external environments, such as the skin, respiratory tract, and oral/gastrointestinal mucosa. Mast cells were discovered by Paul Ehrlich in 1878 and best known for their roles in allergic reactions [12,13]. The classical mast cell activation mechanism through the crosslinking of antigen-specific IgE receptors (FceRI) is a well-studied and a well-known mechanism. On the other hand, non-IgE mediated mast cell activation is less studied and not well established [14,15]. After decades of uncertainty about the receptor responsible for non-IgE mediated life-threatening hypersensitivity reactions, MAS related G protein-coupled receptor X2 (MRGPRX2) was uncovered as the receptor on mast cells in 2006 [15]. Human MRGPRX2 or its mouse orthologue MRGPRB2 is a Class A orphan GPCR expressed on primate’s mast cells [16]. In a recent report in 2019 by IUPHAR/BPS, MRGPRX2 is listed as a Class A orphan GPCR for which preliminary evidence for an endogenous ligand has been published, or for which there exists a potential link to disease [17]. Now, it is known that direct activation of MRGPRX2 causes anaphylactoid shock in humans [1,18]. Unlike other GPCRs, MRGPRX2 can be activated by a plethora of chemicals including cationic amphiphilic drugs (tubocurarine, atracurium, icatibant, ciprofloxacin, and other fluoroquinolone antibiotics), insect venom chemical components (mastoparan and Polistes kinin), antimicrobial peptides (alpha- and beta-defensins and cathelicidins), secreted eosinophil products (eosinophil peroxidase and major basic protein), and neuropeptides (substance P, vasoactive intestinal peptide, neuropeptide Y, somatostatin, and cortistatin) [12,19]. So far, there are no clinical therapies available to cure anaphylactoid reaction. The available treatment such as anti-histamine drugs, adrenaline and mast cell stabilizers, are symptomatic therapies [3,20]. More importantly, the side effects of these available drugs limit their use and are a major concern for clinicians [20].

Genistein is a non-steroidal polyphenol that possesses diverse pharmacological activities. There is evidence for the protective effects of Genistein in inflammation [21,22,23], diabetes [24,25], cancer [26,27] and hypersensitivity reactions [28,29]. In a recent paper, Kim, Dong Hwan et al. reported the anti-inflammatory activity of Genistein on mast cells via inhibiting cytokines and the ERK pathway and proposed potential of Genistein for the treatment of allergic inflammation and anaphylactic shock [30]. However, no study has been conducted on the effect of Genistein on anaphylactoid reaction and its mechanism of action. 

In the present study, we designed a battery of in-vitro, in-silico and in-vivo experiments to evaluate the anti-anaphylactoid activity of Genistein so as to understand its mechanism of action. First, we evaluated the cell toxicity of Genistein on the human mast (LAD-2) and HTLA cells (HEK293 cell line which stably expressing a tTA-dependent luciferase reporter and a β-arrestin2-TEV fusion gene). After assuring that Genistein possesses no toxicity in these cells, we have evaluated its inhibitory actions on the mast cell degranulation and MRGPRX2 activation by β-hexosaminidase and PRESTO-Tango as well as Ca^2+^ flux assays respectively. In addition, via in-silico molecular docking, we predicted both binding affinity and binding site on MRGPRX2. To assess the in-vivo anti-anaphylactoid activity of Genistein, we performed studies utilizing a compound 48/80 induced-anaphylactoid shock mouse model. Our findings taken together demonstrated a therapeutic potential of Genistein as a lead compound in the treatment of anaphylactoid shock.

## 2. Results

### 2.1. No Cytotoxicity of Genistein on Human Mast Cells and HTLA Cells

**Figure 1:**
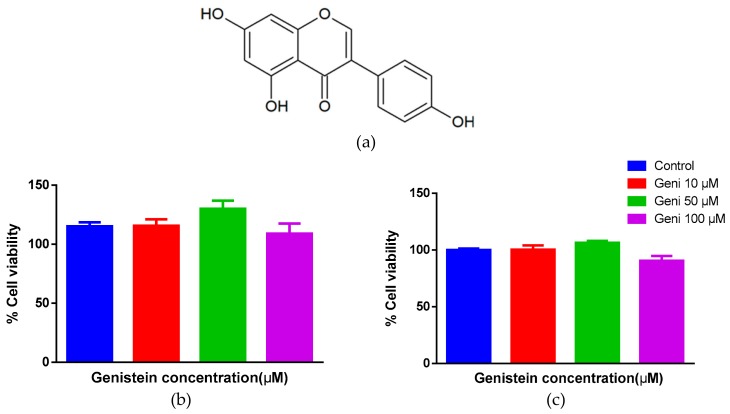
The chemical structure of Genistein (**a**). Cytotoxicity of Genistein on human (LAD-2) mast cells (**b**) and HTLA cells (**c**). Cells (5 × 10^3^ cells/well) were pre-treated with Genistein (0–100 µM) and incubated for 24 h, cell viability was determined by MTT assay. The data are representative of three independent experiments. Each column represents the means ± SEM of three independent experiments.

In order to check if Genistein is toxic, we treated human mast (LAD-2) and HTLA cells (Figure 1a–c) with graded concentrations of Genistein ranging from 0 to 100 µM for 24 h. As shown in Figure 1b,c, treatment of up to 100 µM Genistein did not shown any cell toxicity as compared to the control (<0.1% DMSO), and we have therefore used 50 and 100 μM of Genistein in our subsequent in-vitro experiments.

### 2.2. In-vitro Inhibitory Effect of Genistein on Mast Cell Degranulation, MRGPRX2 Activation and Calcium Flux

**Figure 2:**
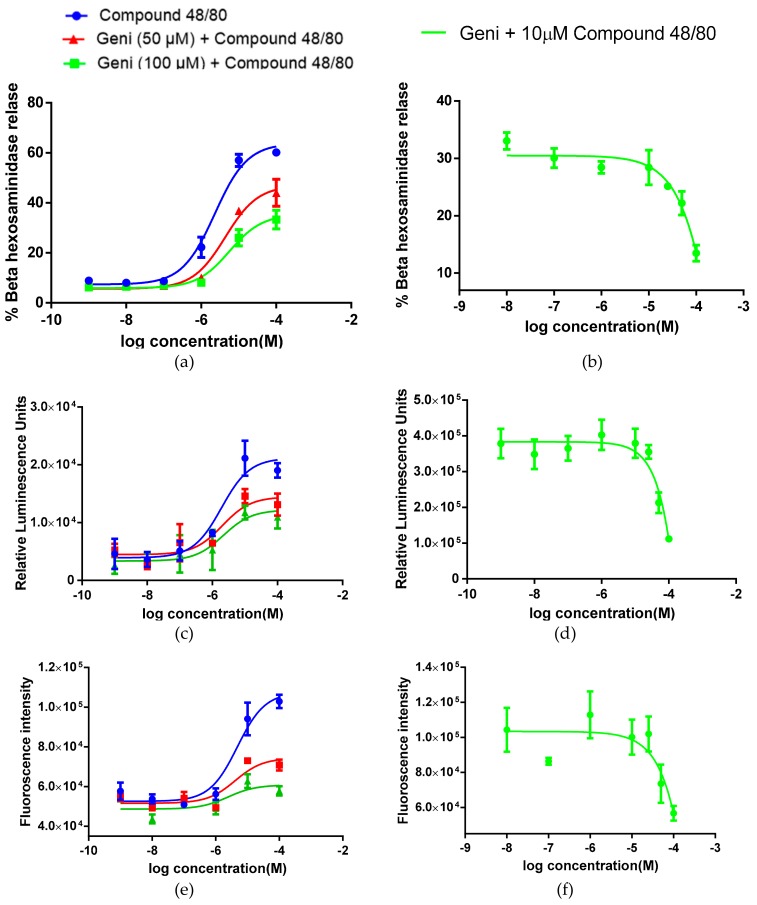
Genistein inhibited human mast cell degranulation. For EC50 shift estimation, we plotted a graph of log concentration (compound 48/80) vs. response (% β-hexosaminidase release) in the presence of buffer, and genistein 50 μM, and 100 μM (**a**). The Dose–Response Curve (DRC) of Genistein for IC50 estimation via mast cell degranulation assay (**b**). Genistein inhibited Mas related GPCRX2 (MRGPRX2) activation measure by PRESTO-Tango assay, DRC of compound 48/80 in the presence of buffer, and Genistein 50 μM, and 100 μM (**c**). The DRC of Genistein for IC50 estimation via MRGPRX2 activation assay (**d**). Genistein inhibited calcium flux in Human LAD-2 mast cells. The DRC of compound 48/80 in the presence of buffer, and Genistein 50 μM, and 100 μM (**e**). IC50 of Genistein was estimated via plotting a graph between log concentration (Genistein) vs. response (fluorescence intensity) (**f**). Each data represents the means ± SEM of three independent experiments.

Mast cell activation leads to the rapid release of pre-formed inflammatory mediators such as β-hexosaminidase, histamine, and inflammatory cytokines. We measured compound 48/80-induced β-hexosaminidase release from LAD-2 mast cell in the presence of 50 and 100 μM Genistein. We found significant changes in EC50 (2.38 × 10^−6^ M vs. 4.48 × 10^−6^ M and 5.76 × 10^−6^ M, respectively) and maximal response (Emax: 63.81% vs. 46.99% and 35.43%, respectively) of compound 48/80 (Figure 2a and Table 1). These data show that Genistein at both doses were capable of reducing efficacy of compound 48/80 in degranulating LAD-2 cells. Also, to verify the antagonizing activity Genistein and to calculate IC50, we performed a Dose–Response Curve (DRC) using graded concentration of Genistein in the presence of 10 μM compound 48/80. Genistein inhibited the activity of compound 48/80 with an IC50 of 2.76 × 10^−5^ M (Figure 2b and Table 1). 

To evaluate the MRGPRX2 antagonistic activity of Genistein, we have performed classical Parallel Receptor-ome Expression and Screening via Transcriptional Output (PRESTO)-tango beta-arrestin assay on MRGPRX2 transfected HTLA cells. In the presence of Genistein 50 μM and 100 μM, the EC50 of compound 48/80 changed (1.98 × 10^−6^ M vs. 2.02 × 10^−6^ M and 2.18 × 10^−6^ M, respectively) and reduced the maximal response (Emax: 21,483 vs. 15,848 and 11,997 respectively, Figure 2c and Table 1). Also, Genistein antagonized the compound 48/80 mediated MRGPRX2 activation with an IC50 of 3.83 × 10^−5^ M (Figure 2d and Table 1). The generation of the calcium signal is crucial for the activation of mast cells and Ca^2+^ is the secondary messenger of MRGPRX2. Genistein inhibited compound 48/80 induced Ca^2+^ flux and shifted EC50 to the right-side dose-dependently. In the presence of Genistein 50 μM and 100 μM the EC50 of compound 48/80 was changed (4.94 × 10^−6^ M vs. 3.15 × 10^−6^ M and 2.74 × 10^−6^ M, respectively) and reduced the maximal response (Emax: 107,462 vs. 74,457 and 60,570 respectively) Figure 2e and Table 1. Moreover, Genistein inhibited the compound 48/80 induced Ca^2+^ flux with an IC50 of 3.16 × 10^−5^ M (Figure 2f and Table 1).

### 2.3. Genistein Binds at a Different Binding Site of MRGPRX2 as of Compound 48/80

**Figure 3:**
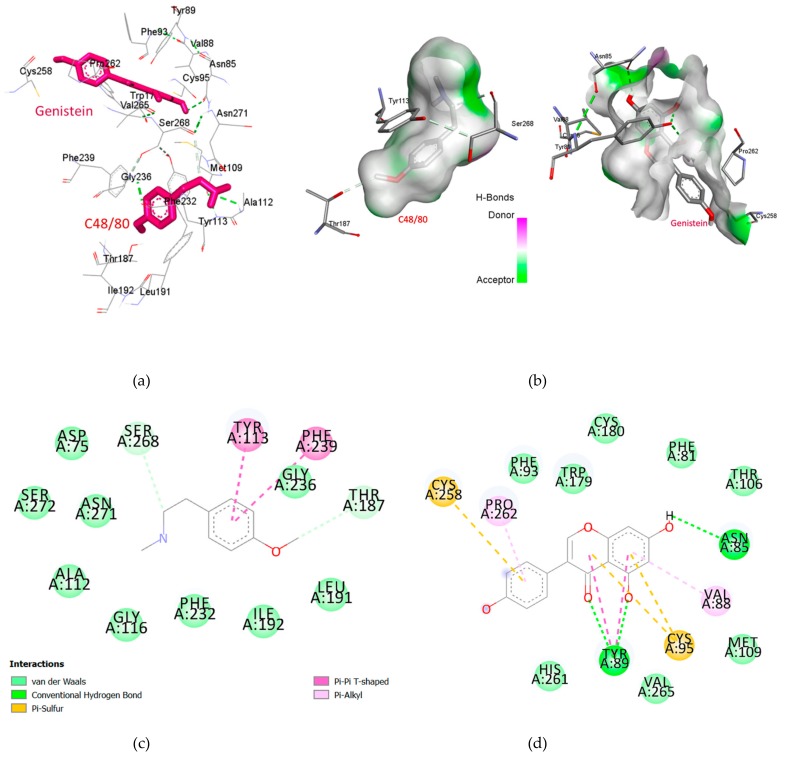
A three-dimensional representation of binding orientation (**a**) and hydrogen bond formation (**b**) of Compound 48/80 and Genistein with MRGPRX2. Two-dimensional representation of interactive amino acid and interaction bond of MRGPRX2 with compound 48/80 (**c**) and Genistein (**d**).

We have used I-TASSER server to model the 3D structure of the MRGPRX2 receptor which was modelled using thermostabilized human C5a anaphylatoxin chemotactic receptor 1 (PDBID 5O9H) as template structure [31]. The template was selected based on three major criteria of sequence identity, phylogenetic classification and C Score. The receptor and template alignment presented sequence homology of 30 percent with query cover of 84%, additionally the C Score was calculated as 1.06 (Table S1 of supplementary information). The 3D model was then validated by Ramachandran plot analysis which shown 99.3% residues in favored and allowed regions with only 2 amino acid residues (i.e., 0.7%) as outliers (Figure S1 of supplementary information). Also, we analyzed the overall quality factor of model (statistics of non-bonded interactions between different atom types) via ERRAT. The normally accepted ERRAT score is >50 for a high quality model [32]. We found an ERRAT score of 96.283 which further validates our model (Figure S2 of supplementary information). A detailed analysis of the interaction between compound 48/80 and Genistein with MRGPRX2 is shown in Figure 3. Based on the docking results, compound 48/80 and Genistein bound at different sites (Figure 3a,b). The highest binding affinities of compound 48/80 and Genistein were −5.8 Kcal/mol and −7.2 Kcal/mol, respectively. The first binding site of the MRGPRX2 receptor bound with 48/80 comprised of Phe239, Tyr113, Thr187, Ser268, Asn271, Gly236, Gly116, Phe232, Leu191, and Ile192. The second binding pocket for Genistein was identified as Cys258, Pro262, Trp179, Asn85, Val88, Cys95, Tyr89, and Thr106. Compound 48/80 interacts with the active amino acid residues via Tyr113 pi-pi stacked, Phe239 pi-pi T shaped, Se268 and Thr187 carbon hydrogen bond, and Gly236 van der Waals bond (Figure 3c). Genistein bounds at a different binding site and interacts with Cys258, and Cys95 via pi-sulfur bonds, Tyr89 and Asn85 hydrogen bonds, Pro262, Val88 pi-alkyl bond, and Tyr89 pi-pi T shaped bond (Figure 3d). The non-competitive binding site of Genistein was consistent with our in-vitro results. This binding information may be useful for the design of structure-based potent derivatives of Genistein.

### 2.4. In-Vivo Inhibitory Effect of Genistein against Local Anaphylaxis Mice Model

**Figure 4:**
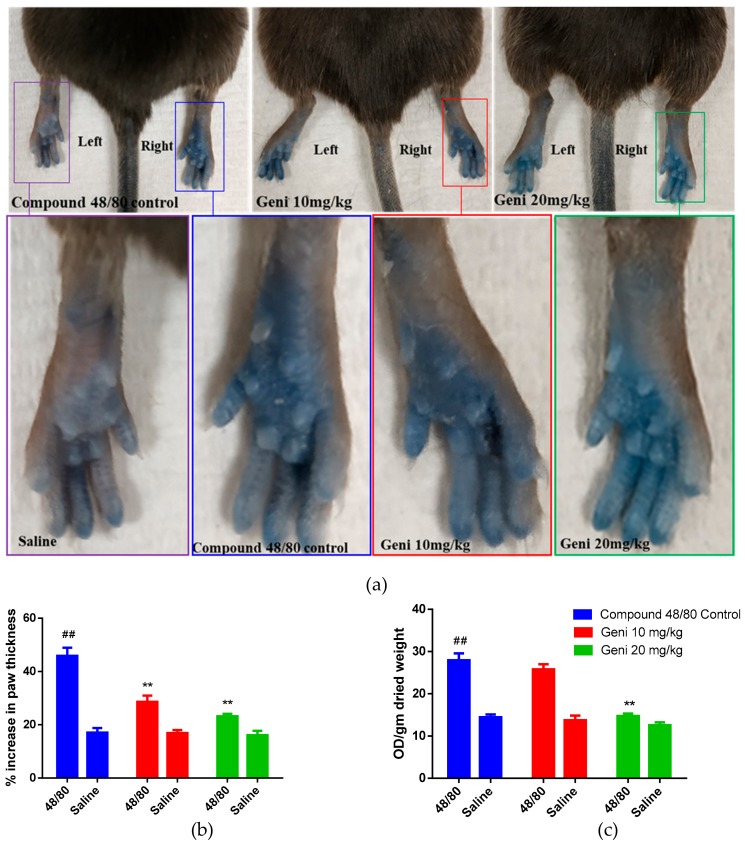
In-vivo evaluation of the anti-anaphylactoid effect of Genistein in local anaphylactoid mice model. Representative images of Evans blue extravasation and cutaneous flare reactions in mice (**a**). Mice were treated with intravenous injection of 10 mg/kg and 20 mg/kg Genistein combined with 50 μL of Evans blue. After 30 min, sub plantar injection of 30 μg/mL compound 48/80 was injected in the right paw and saline in the left paw. Quantification of % increase in paw thickness after 15 min of compound 48/80 injections (**b**). Quantification of Evans blue leakage into the paw after 15 min of compound 48/80 injection (**c**). The data are presented as mean ± SEM (*n* = 6). Ordinary one-way ANOVA followed by Tukey’s multiple comparisons test was used to determine significance in statistical comparisons, and statistical significance was accepted at *p* < 0.05 (** *p* < 0.01 when compared with compound 48/80 control; ## *p* < 0.01 when compared to saline).

To confirm the in-vivo anti-anaphylactoid activity of Genistein, we used a classical animal model of local anaphylactoid shock in mice induced by compound 48/80. Genistein was administered via tail vein injection at a dose of 10 mg/kg, and 20 mg/kg body weight resulted in a dose-dependent decrease in the compound 48/80 induced paw edema. Further, Genistein attenuated the blood vessel leakage tested by Evans blue extravasation from paw tissue (Figure 4b). The hind paws of mice were photographed which demonstrated the inhibition of % paw thickness at both doses of 10 mg/kg and 20 mg/kg as compared to compound 48/80 control (Figure 4a,b). However, on quantification of Evans blue, the significant difference was found only at 20 mg/kg Genistein as compared to compound 48/80 control group (Figure 4c), which shows less potency of Genistein. The in-vivo results supported our in-vitro anti-anaphylactoid activity of Genistein and demonstrated the potential of Genistein as a lead compound for drug development against anaphylactoid shock.

## 3. Discussion

Mast cells play an important role in the immune response by releasing various vasoactive chemokines, cytokines, and functionally diverse proteases [33,34]. Human MRGPRX2 (mouse orthologue MrgprB2) is a Class A orphan GPCR expressed on primates’ mast cells [16]. Human MRGPRX2 binds promiscuously to structurally diverse peptides and small molecules that tend to have basic properties (basic secretagogues), resulting in acute histamine-like adverse drug reactions [35]. Earlier our understanding of mast cell activation was limited to classical IgE Fcε receptor-1 mediated activation [14].

Interestingly in recent years, several US FDA approved drugs such as tubocurarine, atracurium, icatibant, ciprofloxacin, and other fluoroquinolone antibiotics were reported to induce MRGPRX2 [14]. In a recent finding, McNeil et al. [14] reported the MRGPRX2 mediated non-IgE activation of mast cells by these drugs. Therefore, antagonizing MRGPRX2 is a rational therapeutic strategy for the prevention and treatment of anaphylactoid reactions. In recent years, several attempts have been made to target MRGPRX2 for screening antiallergic and anti-anaphylactoid molecules [36,37,38]. Recently some natural compounds such as quercetin [38], saikosaponin A [36], and shikonin [39] have been reported to inhibit mast cell degranulation and inhibit MRGPRX2-induced pseudo allergic reactions. 

Genistein is well known for its anti-inflammatory [21,22,23], anti-diabetic [24,25], and anti-cancer [26,27] activities. In a recent study, Kim, Dong Hwan et al. reported the potential anti-allergic and anti-inflammatory activity of Genistein on mast cells via inhibiting cytokines and the ERK pathway [30]. However, there is no direct evidence on the effect of Genistein on mast cells mediated anaphylactoid reaction and its mechanism of action. In the present study, we evaluated the in-vitro and in-vivo anti-anaphylactoid activity of Genistein and its mechanism of action. 

In the first experiment, we have evaluated the toxicity of Genistein in human mast cells and HTLA cells through MTT assay. The MTT assay is a colorimetric assay for measuring cell metabolic activity and safety of drug-like molecules and widely used for screening of cell cytotoxicity [40]. Genistein demonstrated no toxicity up to 100 µM concentration in both cell lines. Based on these results, we have used a maximum concentration of 100 µM in our further experiments. Human LAD-2 mast cells were used to evaluate the inhibitory activity of Genistein against compound 48/80 induced mast cell degranulation [41]. Mast cells are granulated immune cells, storing several pre-synthesized inflammatory mediators [42]. Once mast cells get activated via endogenous or exogenous ligands, they immediately release the inflammatory mediators into surrounding tissues. Compound 48/80 is a well-known MRGPRX2 agonist in experimental pharmacology which activate MRGPRX2 and induce mast cell degranulation [43,44,45]. Genistein dose-dependently shifted the compound 48/80′s mast cell degranulation EC50 to the right side and lowered the Emax. Also, at higher concentrations, Genistein completely blocked the compound 48/80 activity.

To understand the mechanism and receptor involved in mast cell degranulation inhibitory activity of Genistein, we used MRGPRX2 transfected HTLA cell lines. Genistein antagonized the compound 48/80-induced MRGPRX2 activations and shifted the DRC of compound 48/80 to the right side. The EC50 shift and Emax lowering the effect of Genistein were concentration-dependent (both doses of 50 µM and 100 µM). At higher concentrations, Genistein completely inhibited the compound 48/80 induced MRGPRX2 activations.

Moreover, to verify mast cell degranulation and PRESTO-tango assay results, we measured the effect of Genistein on MRGPRX2 secondary messenger (Ca^2+^ flux). The generation of the calcium signal is crucial for the activation of mast cells. This signal results from MRGPRX2 mediated activation of phospholipase C and the associated production of inositol 1,4,5-trisphosphate (IP3), which induces the release of Ca^2+^ from stores in the Endoplasmic Reticulum and Golgi through Ca^2+^ conducting IP3-receptors [31]. Activation of MRGPRX2 via compound 48/80 results in mobilization of Ca^2+^, followed by mast cell degranulation and inflammatory reactions. We evaluated the MRGPRX2 antagonistic activity via testing Ca^2+^ flux inhibitory potential of Genistein on LAD-2 mast cells. Genistein antagonized the compound 48/80 induced Ca^2+^ flux and shifted the DRC of compound 48/80 to the right side in a concentration-dependent manner. Also, at higher concentrations, Genistein completely abolished the Ca^2+^ flux. Overall, Genistein has shown potential as a lead compound for the treatment of anaphylactoid shock with an IC50 of approximately 25–35 µM (in different bioassays). As the potency is low, more efforts are needed to improve the potency of Genistein via the chemistry program [46]. Collectively, the in-vitro results are very encouraging and provide direct evidence of MRGPRX2 antagonism by Genistein. In the presence of Genistein, we found a right-hand shift in the EC50 and a decrease in the Emax of compound 48/80 DRC. These results indicate the non-competitive inhibition of MRGPRX2, where Genistein is not competing for the same active site with compound 48/80. 

Interestingly, in our in-silico results, we found that compound 48/80 and Genistein are not binding at the same binding site on MRGPRX2, which supports our in-vitro findings of non-competitive antagonism. To understand the binding affinity, the binding site of Genistein on MRGPRX2, we have performed docking experiments. Docking is a powerful tool for the prediction of binding affinity and interaction of a new drug-like molecule with a receptor [47]. The scoring function of molecular docking has been used to understand the binding affinity and interaction between drug and receptor [47,48]. Unfortunately, the crystallographic information of MRGPRX2 is not yet available on RCSB Protein Data Bank. The limited structural information reduces the binding site information and experimental data interpretation. However, the availability of online 3D structure prediction software such as I-TASSER (https://zhanglab.ccmb.med.umich.edu/I-TASSER/) helps in the understanding of ligand binding and molecular interactions [49]. We modelled the 3D structure of MRGPRX2 using I-TASSER server and validated by Ramachandran plot analysis and ERRAT score [32,50]. Molecular docking studies suggested that the Genistein binds at a different binding site then compound 48/80 and interacted with Cys258, and Cys95 via pi-sulfur bonds, Tyr89 and Asn85 hydrogen bonds, Pro262 and Val88 pi-alkyl bond, and Tyr89 pi-pi T shaped bond. The amino acid drug interaction map unveiled that the numerous van der Waals, carbon–hydrogen, Pi alkyl, and pi-pi T shaped are the critical force for higher binding affinity of Genistein then compound 48/80. Therefore, Genistein has shown better binding energy for MRGPRX2, and it may be considered as a considerable MRGPRX2 antagonist. 

Based on the earlier studies, we selected 10 mg/kg and 20 mg/kg body weight doses of Genistein for in-vivo assessment of anti-anaphylactoid activity in mice [51,52]. Due to the release of stored inflammatory cytokines and proteases, edema or swelling of surrounding tissue is the very first symptom of local anaphylactoid shock. Genistein at a dose of 20 mg/kg significantly inhibited the hind paw swelling and edema. Also, we have quantitatively measured the Evans blue extravasation in the paw tissue to evaluate the blood vessels leakage inhibitory activity of Genistein. Evans blue is an albumin-binding dye used to measure the blood vessel’s permeability in mice [53]. In normal physiologic conditions, blood vessels, endothelial cells are impermeable to albumin. Therefore, Evans blue bound albumin remains restricted within blood vessels. However, in pathologic conditions such as local anaphylactoid reaction induced by compound 48/80, the endothelial cells lose their close contacts, and the endothelium becomes permeable to small proteins such as albumin [53]. The albumin-bound Evans blue therefore leaked out from the blood vessels, which was measured by simple visualization as well as by quantifying the Evans blue per gram of tissue. Genistein at a dose of 20 mg/kg significantly decreased the Evans blue extravasation which in turn indicates the protective effect of Genistein against compound 48/80 induced local anaphylactoid reactions. 

The present study has demonstrated in-vitro and in-silico evidence for the interaction of the Genistein with MRGPRX2. However, use of MRGPRB2 knockout mouse models can provide additional support for development of Genistein or its derivatives as lead molecule in the future research. Also, for a better understanding of MRGPRX2 mediated anaphylactoid shock and novel drug development, there is a need for a more in-depth understanding of the binding site and signal pathways of MRGPRX2, along with clinical and epidemiologic studies on anaphylactoid shock. Our findings will encourage researchers to study the MRGPRX2 pathway as well as novel drug development for non-IgE, mediated anaphylactoid shock and other immune disorders.

## 4. Materials and Methods 

### 4.1. Drugs and Reagents

Genistein (>98.0% purity) was purchased from TCI Development Co., Ltd. (Shanghai, China), Compound 48/80, p-nitrophenyl N-acetyl-β-d-glucosamide (PNAG) and triton X-100 were purchased from Sigma-Aldrich Co., LLC. (Shanghai, China). Fluo-4 NW calcium assay kit was purchased from Thermo Fisher Scientific (Eugene, OR, USA). All other chemicals were of chemical grade and purchased from commercial sources. 

### 4.2. Drug Preparation

Genistein was dissolved in dimethyl sulfoxide (DMSO) (Sigma, St. Louis, MO, USA) and diluted to the desired concentration in buffer or media (final DMSO concentration 0.1% *v*/*v* for cell culture experiments). For in-vitro experiments, an equal amount of DMSO was added to the control samples (medium only). For the in-vivo experiment, 30 mg/mL stock of Genistein (in sterile 100% DMSO) was diluted with normal saline.

### 4.3. Cell Lines 

Human mast cell Laboratory Allergic Disease 2 (LAD-2) cell line was provided by Professor LAU H. Y. Alaster (The Chinese University of Hong Kong, Hong Kong, China SAR). LAD-2 cells were routinely grown in StemPro-34 medium supplemented with 10 mL/l StemPro nutritional supplements, penicillin (1:100), streptomycin (1:100), 2 mmol/l glutamine and 100 ng/mL human stem cell factor and incubated at 37 °C in 5% CO_2_ incubator. Hemi-depletions of media were performed weekly and cell proliferation was examined by measuring total numbers of cells weekly [54]. HTLA cells (HEK293 cell line which stably expressing a tTA-dependent luciferase reporter and a β-arrestin2-TEV fusion gene) was kindly provided by Dr. Leo T.O. Lee, (University of Macau, Taipa, Macau, China) and routinely maintained in Dulbecco’s Modified Eagle Medium (DMEM) supplemented with 10% Fetal Bovine Serum (FBS), 2 μg/mL puromycin and 100 μg/mL hygromycin B in a humidified atmosphere at 37 °C in 5% CO_2_ incubator.

### 4.4. Animals

C57BL/6 adult male mice, 7–8 weeks old, were purchased from the Laboratory Animal Unit (LAU) of the University of Hong Kong (AAALAC International accredited). Mice were housed under a 24 h light/dark cycle, with food and water ad libitum. This study was conducted in strict accordance with the recommendations stated in the Guide for the Care and Use of Laboratory Animals of the National Institutes of Health. The experimental protocols for the mouse experiment were approved by the Committee on the Use of Live Animals in Teaching and Research (CULATR 5125-19) of the University. All animal procedures were performed under ketamine/xylazine anesthesia.

### 4.5. Cell Toxicity Assay

Cell cytotoxicity was determined using the MTT assay using standard protocol [40,55] with a slight modification. Human LAD-2 mast cells and HTLA cells were seeded into 96-well plates (2.2 × 10^4^ cells/well/90 µL) and allowed to adhere for 24 h (HTLA cells only) at 37 °C in a 5% CO2 incubator. LAD-2 cells are non-adherent cells; therefore, they do not need 24 h of incubation. After 24 h of incubation (HTLA cells), the culture medium was replaced with a fresh medium and cells were treated with 10 µL of 10X stock concentrations (0–100µM) of the Genistein for 24 h at 37 °C in a 5% CO2 incubator. After 24 h of incubation, an equal volume (100 μL for LAD-2 cells) or 20 μL (for HTLA cells) of MTT solution (5 mg/mL in phosphate buffer solution) was added to each well and plate was incubated for 4 h at 37 °C in 5% CO2 incubator. For LAD-2 cells, the plate was centrifuged at 500× *g* for 5 min and the media was aspirated, while for HTLA cells medium was aspirated directly. The formed formazan crystals were solubilized by adding 150 μL of MTT solvent (4mM HCL + 0.1% NP-40 in isopropanol) per well in a 4 °C shaker for 15 min. Finally, the intensity of the dissolved formazan crystals (purple color) was quantified using the Victor 4X plate reader (PerkinElmer) at 540 nm. Per cent cell viability was calculated by comparing the % cell viability with control cells using the Graph Pad Prism software (6.01, Graphpad Software Inc, San Diego, CA, USA).

### 4.6. Mast Cell Degranulation (β-Hexosaminidase) Assay

Human LAD-2 mast cells were seeded at 2 × 10^5^ cells/well/80 μL in a 96-well plate. A 10X stocks of Genistein was prepared in HEPES buffer (HEPES (10 mM), NaCl (137 mM), KCl (2.7 mM), Na_2_HPO_4_·7H_2_O (0.4 mM), glucose (5.6 mM), CaCl_2_·2H_2_O (1.8 mM), MgSO_4_·7H_2_O (1.3 mM), bovine serum albumin (0.04%), pH 7.4). Then, the 10 μL of 10X Genistein stock (final concentration was 50 μM and 100 μM) or buffer was added to cells and incubated at 37 °C for 30 min without CO_2_. Further, 10 μL of graded concentrations of 10X stock of compound 48/80 or buffer was added to each well and cells were incubated at 37 °C for an additional 30 min without CO_2_. For evaluating Genistein DRC, 10 µL of graded concentrations of 10X stock of Genistein were added, cells were incubated for 30 min at 37 °C without CO_2_. Then 10 µL of 10X compound 48/80 (final concentration in each well was 10 µM) was added to each well and cells were incubated at 37 °C for an additional 30 min without CO_2_. The plate was spun at 450× *g*, at room temperature for 5 min to stop the reaction and to ensure the cells are sedimented to the base of the wells. Fifty microliters of supernatant was aliquoted to a new 96-well plate via tilting the plate to an angle of 45°. The cells were further lysed with 100 μL of 0.1% Triton X-100 and 50 μL of lysate was aliquoted to a new 96-well plate. The substrate PNAG solution (1.3 mg/mL) was prepared in 0.1 M citric acid/sodium citrate buffer (pH 4.5, 24.087 g/L Sodium Citrate dihydrate and 3.471 g/L Citric Acid) and 50 μL of PNAG was added to each well of supernatant and lysate. The plate was incubated for 90 min at 37 °C (without CO2) and then 50 μL of 0.4 M Glycine buffer was added into each well. The appearance of the yellow color indicates the extent of the β-hexosaminidase release. The plate was measured at 405 nm using Victor 4X plate reader (PerkinElmer, Waltham, MA 02451 USA) and percentage of β-hexosaminidase release was calculated by using the following formula;

% degranulation = OD at 405 of supernatant/(OD at 405 of supernatant + OD at 405 of lysate) × 100.

DRC was plotted via Graph Pad Prism using a non-linear regression (curve fit) model of log (agonist or antagonist) vs. response (three parameters).

### 4.7. MRGPRX2 Activation Assay (PRESTO-Tango Assay)

MRGPRX2 activation was measured by PRESTO-tango assay, according to Kroeze, Wesley K et al. with slight modification [56]. HTLA cells were maintained in DMEM supplemented with 10% FBS, 2 μg/mL puromycin and 100 μg/mL hygromycin B in a humidified atmosphere at 37 °C in 5% CO_2_ incubator. For transfection, cells were seeded at 1 × 10^6^ cells per 100 mm cell culture dish (day 1). The following day (day 2), cells were transfected using the Lipofectamine transfection reagent (Thermo Fischer Scientific). On day 3, transfected cells were transferred at 2 × 10^4^ cells/well/80 μL of the medium into 96-well white clear-bottom cell culture plates (PerkinElmer Life Science). On day 4, 10 μL of 10X Genistein (final concentration of 50 μM and 100 μM) or buffer (20 mM HEPES in 1x HBSS at pH 7.4), were added to each well and plate was incubated at 37 °C in 5% CO_2_ incubator for 4 h. After 4 h, 10 μL of 10X stock graded concentrations of compound 48/80 or buffer was added to each well and plate was incubated for another 14 h at 37 °C in 5% CO_2_ incubator. For evaluating Genistein DRC, 10 µL graded concentrations of 10X stock of Genistein were added to each well for 4 h. After 4 h, 10 μL of 10X compound 48/80 or buffer (final concentration in each well was10 μM) was added to each well and plate was incubated for another 14 h at 37 °C in 5% CO2 incubator. On day 5, medium and drug solutions were removed from the wells (by aspiration), and 30 μL per well of Bright-Glo solution (Promega) was added to each well with a multichannel pipette. After incubation for 5 min at room temperature, luminescence was measured in a Victor 4X plate luminescence counter (PerkinElmer). Results in the form of RLU (relative luminescence units) were exported into Excel spreadsheets, and Graph Pad Prism was used for the analysis of data. DRC was plotted via Graph Pad Prism using a non-linear regression (curve fit) model of log (agonist or antagonist) vs. response (three parameters).

### 4.8. Intracellular Ca^2+^ Flux Assay

The Ca^2+^ flux was measured in human LAD-2 mast cells by using the Fluo-4 NW calcium assay kit and the protocol according to the manufacturer. In short, LAD-2 cells were plated in a 96-well black well plate (SPL Life Sciences) at a density of 1 × 10^4^ cells/well/40µL in assay buffer and incubated for 1 h at 37 °C incubator. After 1 h, 10 µL of 10X stock Genistein (final concentration was 50 µM and 100 µM) or buffer followed by 40 µL of the 2X dye loading solution were added to each well. The plate was incubated for 30 min at 37 °C in 5% CO2 incubator and then at room temperature for an additional 30 min. Then 10 µL of graded concentrations of 10X stock of compound 48/80 or buffer was added to respective wells. For evaluating Genistein DRC, 10 µL of graded concentrations of 10X stock of Genistein was added, cells were incubated for 30 min at 37 °C in 5% CO2 incubator and then at room temperature for additional 30 min. Then 10 µL of 10X compound 48/80 (final concentration in each well was 10 µM) was added to the cells. Fluorescence was measured via setting excitation at 494 nm and emission at 516 nm using Victor 4X plate reader (PerkinElmer). DRC was plotted via Graph Pad Prism using a non-linear regression (curve fit) model of log (agonist or antagonist) vs. response (three parameters). 

### 4.9. Molecular Docking of Genistein

The crystallographic information of MRGPRX2 is not yet available on RCSB Protein Data Bank [57], which limits the experimental data interpretation and novel mechanistic hypotheses generations. We used I-TASSER for the prediction of MRGPRX2 3D protein structure [58]. We selected the template based on three major criteria of sequence identity, phylogenetic classification and C score. The selected templates model was crystal structure of thermostabilized human C5a anaphylatoxin chemotactic receptor 1 (PDBID 5O9H). Along with high sequence similarity calculated by Z score, the C5a receptor model and MRGPRX2 receptors belong to same class of G protein-coupled receptors indicating close phylogenetic relationship. The selected model was evaluated by Ramachandran plot analysis, which presented 99.3% residues in the favored and allowed region with only 2 amino acid residues (i.e., 0.7%) as outliers [50]. These two amino acids (Arg141 and Leu198) were confirmed as not being at the compound binding sites of the receptor. Ramachandran plot calculation was performed by the RAMPAGE: Assessment of Ramachandran Plot and Schrodinger Maestro. Further, the model was validated via ERRAT score. We found a 96.283 ERRAT score, which shows it is a high quality model [32]. Molecular docking was performed by using Auto Dock Vina [59]. The protein and ligand optimization such as removal of all the crystallographic water molecules, the addition of polar hydrogens and Gasteiger charges was done by using AutoDock4 tools (ADT4) [60], Avogadro [61] and Chimera [62]. The atomic coordinates of Genistein and compound 48/80 were drawn by ACD/ChemSketch (freeware) and saved as an MOL file. All three ligands were optimized by an advanced molecule editor and visualizer software Avogadro. By using the auto-optimization tool of Avogadro which provides an interactive interface, Universal Force Field (UFF), and Steepest descent algorithm all ligands were optimized and saved as a PDB file. The saved PDB files of ligands were loaded in ADT4 for changing to PDBQT format and files were saved as PDBQT file. Molecular docking simulations were performed using Auto Dock Vina’s Broyden–Fletcher–Goldfarb–Shanno (BFGS) search algorithm. The visualization protein–ligand interactions were done by PyMol [63], maestro [64] and Discovery studio visualizer [65]. 

### 4.10. In-Vivo Anti-Anaphylactoid Activity

Young adult male mice (C57/BL/6N aged 6–8 weeks old, *n* = 6/group) were anesthetized via intraperitoneal injection of ketamine and xylazine. Then 50 μL (irrespective of the body weight) of 12.5% Evans blue in saline along with 10 mg/kg and 20 mg/kg of Genistein was injected via the tail vein. Before injection, a Vernier caliper was used to measure the thickness of both the paws. Thirty minutes later, 10 μL of 30 μg/mL compound 48/80 was injected into one paw, and saline was injected into the other paw as a negative control. Fifteen minutes later, the paw thicknesses were measured again and documented. Mice were euthanized by cervical dislocation under anesthesia, and the paw tissues were collected in a 1.5 mL Eppendorf tube, dried at 50 °C for 24 h, and weighed separately. Evans blue dye was extracted by adding 500 μL of formamide to each tissue sample and incubating at 50 °C for 24 h. The supernatant was aliquoted equally into 96-well plates (200 μL/well, duplicate), and the OD value at 495 nm was measured using Victor 4X plate reader (PerkinElmer). A graph was plotted by calculating the % increase in paw thickness using Graph Pad Prism. OD value was then normalized to per gram dried weight of each paw, and a graph was plotted between OD/gm weight of paw tissue vs. group using Graph Pad Prism.

### 4.11. Statistical Analysis

All data are shown as means ±standard error of the mean (SEM). The graphs between groups was plotted using Prism 7.0 software (GraphPad Software Inc.). The data were analyzed using analysis of variance (ANOVA), two-tailed tests, followed by Tukey’s multiple comparisons test. A value of *p* < 0.05 was considered to be significant.

## 5. Conclusions

In conclusion, our study demonstrated the protective effect of Genistein against compound 48/80 induced anaphylactoid shock via inhibiting MRGPRX2. Genistein demonstrated a non-competitive antagonistic activity that was evidenced by mast cells degranulation assay, PRESTO-tango and Ca^2+^ flux assay. Also, the molecular docking results supported our wet lab experimental results, which opens up the possibility for further research on Genistein as a lead compound and structure-based drug design for the development of potent small molecular MRGPRX2 inhibitors for the treatment of anaphylactoid shock.

## Figures and Tables

**Table 1:** The effect of Genistein on the EC50 and maximal response (Emax) of compound 48/80 and IC50 of Genistein via different in-vitro assays. For the EC50 and Emax, estimation cells were treated with buffer or Genistein (50 µM and 100 µM) followed by graded concentrations of compound 48/80. For IC50 estimation, cells were treated with graded concentrations of Genistein followed by 10 µM of compound 48/80.

In-vitro assays	EC50			E Max			IC50
	Graded Concentration of Compound 48/80						Genistein +10 µM Compound 48/80
	Buffer	Genistein (50 µM)	Genistein (100 µM)	Buffer	Genistein (50 µM)	Genistein (100 µM)	
β-Hex	2.38 × 10^−6^ M	4.48 × 10^−6^ M	5.76 × 10^−6^ M	63.81	46.99	35.43	2.76 × 10^−5^ M
PRESTO-tango	1.98 × 10^−6^ M	2.02 × 10^−6^ M	2.18 × 10^−6^ M	21,483	15,848	11,997	3.83 × 10^−5^ M
Ca^2+^ flux	4.94 × 10^−6^ M	4.07 × 10^−6^ M	3.61 × 10^−6^ M	107,462	74,457	60,570	3.16 × 10^−5^ M

## Supplementary Materials

The following are available online. Figure S1: Ramachandran plot depicting 99.3% residues in favored and allowed region with only 2 amino acid residues as outlier. The calculation was performed by the RAMPAGE: Assessment of Ramachandran Plot. Figure S2: ERRAT score helps in analysing statistics of non-bonded interactions between different atom types. The model presented overall quality factor as 96.283. The calculation was made by the help of SAVES server, Table S1: Depicts the sequence similarity, Z score, C score and ERRAT quality factor of selected template.
