# Supplementary material for: Protective Effect of Genistein against Compound 48/80 Induced Anaphylactoid Shock via Inhibiting MAS Related G Protein-Coupled Receptor X2 (MRGPRX2)
Source: Molecules. 2020 Feb 25;25(5):1028. doi: 10.3390/molecules25051028 (PMC7179155; doi:10.3390/molecules25051028)
Supplement: Supplementary file 1 [file molecules-25-01028-s001.pdf]

Protective effect of Genistein against compound 48/80 induced anaphylactoid shock via inhibiting MAS related G protein-coupled receptor X2 (MRGPRX2)

Mukesh Kumar[1], Kailash Singh[1], Karthi Duraisamy[1], Ahmed A Allam[2], Jamaan Ajarem[3], Billy Kwok Chong CHOW[1*]

Figure S1.

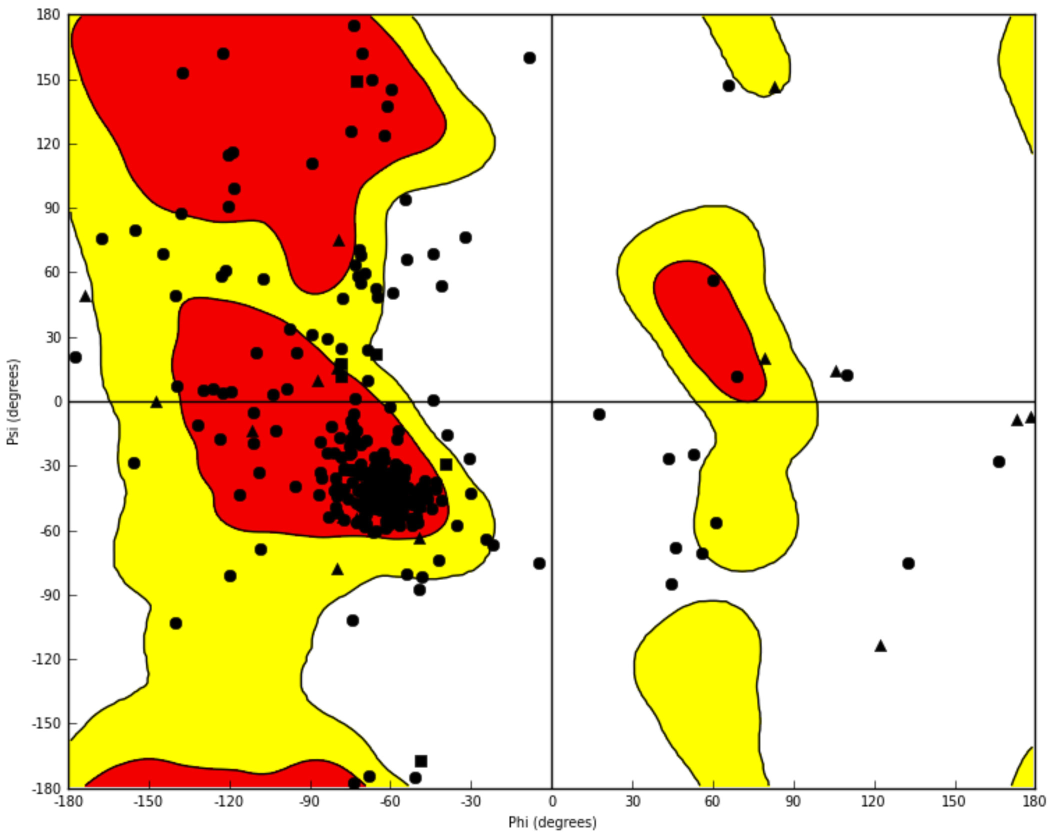

Figure S1. Ramachandran plot depicting 99.3% residues in favored and allowed region with only 2 amino acid residues as outlier. The calculation was performed by the RAMPAGE: Assessment of Ramachandran Plot.

Figure S2.

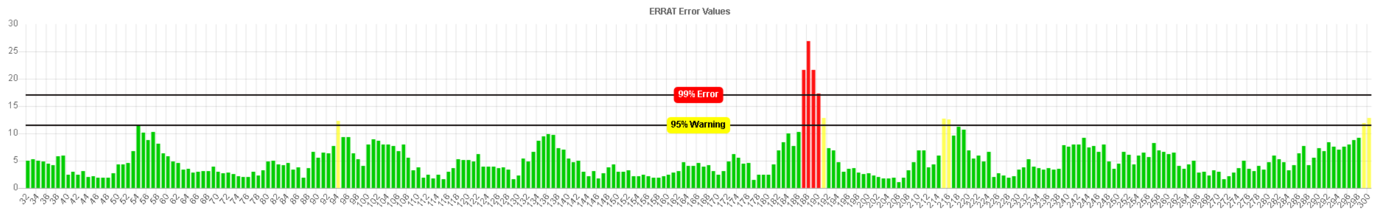

Figure S2. ERRAT score helps in analyzing statistics of non-bonded interactions between different atom types. The model presented overall quality factor as 96.283. The calculation was made by the help of SAVES server.

Table S1: Depicts the sequence similarity, Z score, C score and ERRAT quality factor of selected template

Sequence similarity	Query Coverage	Z Score	C Score	ERRAT Quality Factor
30%	84%	3.01	1.06	96.283
